# A single amplified genome catalog reveals the dynamics of mobilome and resistome in the human microbiome

**DOI:** 10.1186/s40168-024-01903-z

**Published:** 2024-10-02

**Authors:** Tetsuro Kawano-Sugaya, Koji Arikawa, Tatsuya Saeki, Taruho Endoh, Kazuma Kamata, Ayumi Matsuhashi, Masahito Hosokawa

**Affiliations:** 1 bitBiome, Inc., 513 Wasedatsurumaki-Cho, Shinjuku-Ku, Tokyo, 162-0041 Japan; 2 Department of Life Science and Medical Bioscience, Waseda University, 2-2 Wakamatsu-Cho, Shinjuku-Ku, Tokyo, 162-8480 Japan; 3 Computational Bio Big-Data Open Innovation Laboratory, National Institute of Advanced Industrial Science and Technology, 3-4-1 Okubo, Shinjuku-Ku, Tokyo, 169-8555 Japan; 4 Institute for Advanced Research of Biosystem Dynamics, Waseda Research Institute for Science and Engineering, 3-4-1 Okubo, Shinjuku-Ku, Tokyo, 169-8555 Japan; 5 Research Organization for Nano and Life Innovation, Waseda University, 513 Wasedatsurumaki-Cho, Shinjuku-Ku, Tokyo, 162-0041 Japan

## Abstract

**Background:**

The increase in metagenome-assembled genomes (MAGs) has advanced our understanding of the functional characterization and taxonomic assignment within the human microbiome. However, MAGs, as population consensus genomes, often aggregate heterogeneity among species and strains, thereby obfuscating the precise relationships between microbial hosts and mobile genetic elements (MGEs). In contrast, single amplified genomes (SAGs) derived via single-cell genome sequencing can capture individual genomic content, including MGEs.

**Results:**

We introduce the first substantial SAG dataset (bbsag20) from the human oral and gut microbiome, comprising 17,202 SAGs above medium-quality without co-assembly. This collection unveils a diversity of bacterial lineages across 312 oral and 647 gut species, demonstrating different taxonomic compositions from MAGs. Moreover, the SAGs showed cellular-level evidence of the translocation of oral bacteria to the gut. We also identified broad-host-range MGEs harboring antibiotic resistance genes (ARGs), which were not detected in the MAGs.

**Conclusions:**

The difference in taxonomic composition between SAGs and MAGs indicates that combining both methods would be effective in expanding the genome catalog. By connecting mobilomes and resistomes in individual samples, SAGs could meticulously chart a dynamic network of ARGs on MGEs, pinpointing potential ARG reservoirs and their spreading patterns in the microbial community.

Video Abstract

**Supplementary Information:**

The online version contains supplementary material available at 10.1186/s40168-024-01903-z.

## Introduction

The intimate connection between humans and their associated microbiomes has received significant research attention given its crucial ramifications, including its influence on human health, disease progression, and treatment responses [1–5]. The advent of metagenomics has provided unprecedented insights, particularly by unlocking data from uncultured microbes. Genome catalogs such as the Unified Human Gastrointestinal Genome Catalogue [6–11] stand out in this endeavor, curating comprehensive microbial genomes from microbial communities. A number of metagenome-assembled genomes (MAGs) are registered in these catalogs, but some biological information may be missing from the genome sequences.

Notably, metagenomics, in its principle of assembling and aggregating similar sequences, struggles to render MAGs that link information on highly conserved sequences, such as rRNA genes, and mobile genetic elements (MGEs), including plasmids and phages. The limitations of metagenomics have been previously reported [12–15]. For instance, only 7% of even highly complete human gut MAGs yielded 16S rRNA genes [16]. Furthermore, another study reported low presence rates of MGEs in MAGs (38–44% for genomic islands and 1–29% for plasmids) and a complete lack of virulence genes and antibiotic resistance genes (ARGs) in plasmids [17]. Another challenge for metagenomics is distinguishing whether sequence reads are from intact microbes or free fragment DNA in the sample. Because of this limitation, metagenomics is not well suited for assessing microbial transfer and survival between different environments.

Single-cell genome sequencing has emerged as a potential avenue to overcome these challenges by constructing single amplified genomes (SAGs) from individual microbial strains, including highly conserved genes and MGEs. While this method theoretically reveals cell-to-cell variation, its practical realization depends on the evolution of supporting technologies. Despite advancements in high-throughput single-cell genome sequencing technologies, such as droplet barcoding sequencing [18–20] and their ability to concurrently acquire tens of thousands of SAGs, several challenges persist. Due to the low completeness of SAGs produced by those technologies, the in silico integration of multiple SAGs was generally required to construct quality genomes. This process results in the recovery of a few representative genomes from tens of thousands of low-quality SAGs. It risks obscuring strain heterogeneity information, such as the relationship between the SAGs and MGE or ARG.

We have developed a high-quality, high-throughput single-cell genome sequencing technology, named SAG-gel [21, 22], which enables the simultaneous generation of hundreds or thousands of SAGs. It can obtain SAGs above medium-quality without pooling the SAGs to generate consensus genomes. This advantage is attributed to efficient whole-genome amplification and deep single-cell sequencing by coupling in-gel and well-formatted reactions. Thus far, we have applied our method to various microbiomes, not only from human-associated samples but also from environmental samples, enabling us to reach novel implications such as strain heterogeneity, including MGEs [14, 21–24].

In this study, we aimed to explore the human microbiome at the single-cell level, focusing on the oral and fecal microbiomes, which are linked in the body and are closely associated with human health and disease. We present the bbsag20 dataset, which comprises 17,202 SAGs of medium-quality and above derived from the human oral and gut microbiomes of Japanese individuals using SAG-gel technology. This dataset, being one of the largest human oral and gut bacterial SAGs, offers a rich resource for exploring the intricate dynamics of the microbiomes, mobilomes, and resistomes. We uncovered compelling evidence of oral bacterial translocation to the gut at the cellular level. Furthermore, we elucidated unexpectedly broad host ranges of plasmids and phages and detailed individual differences in ARG and MGE prevalence and their networks.

## Results

### Comparison of genomes obtained by metagenomics and single-cell genomics

**Fig. 1:**
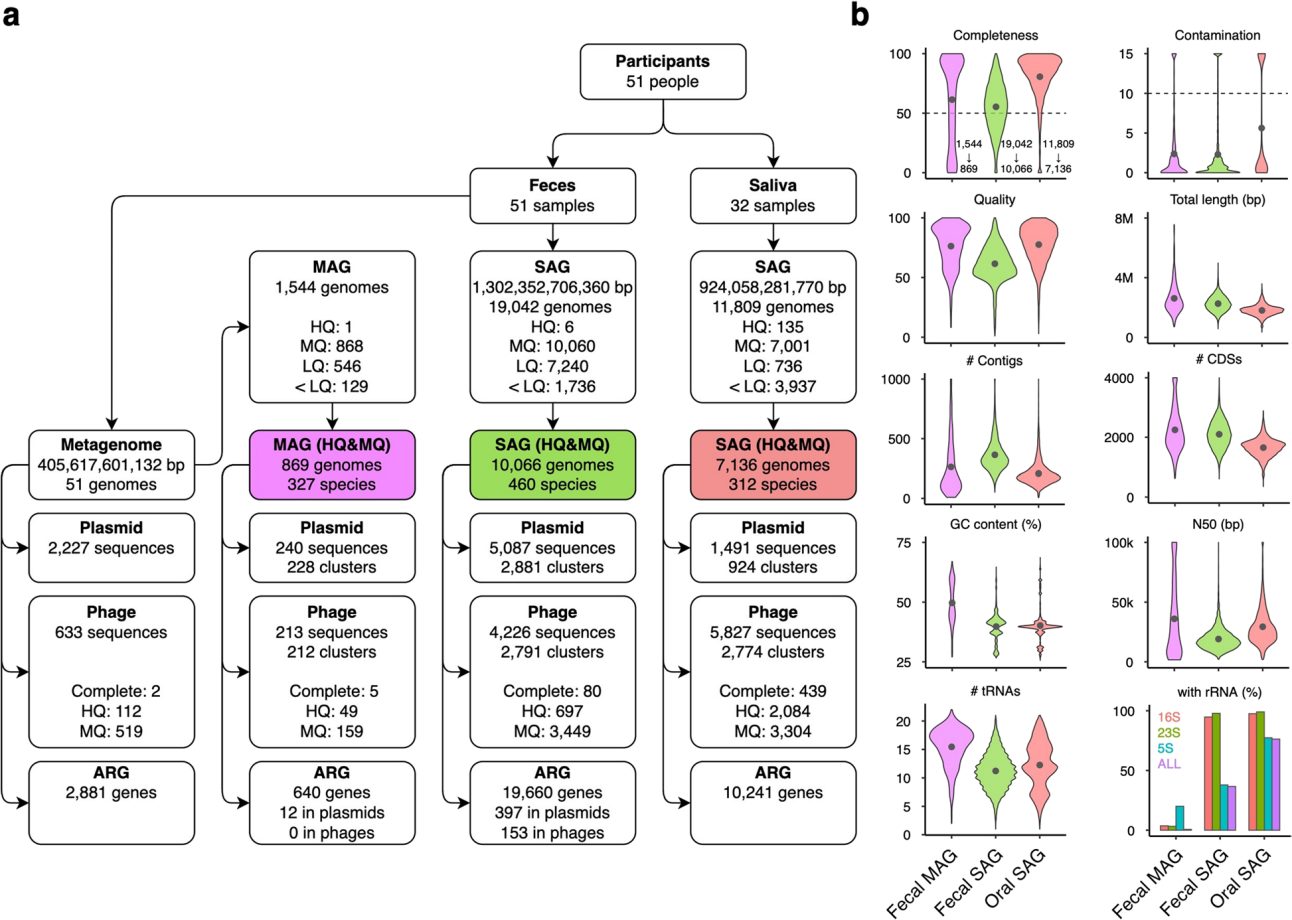
Overview of the Single Amplified Genome catalog bbsag20 for human oral and fecal bacteria. **a** Overview of samples, assembled genomes, MGEs, and ARGs in the bbsag20 dataset. SAGs and MAGs were categorized as high-quality (HQ), medium-quality (MQ), or low-quality (LQ). **b** Assembly statistics for both SAGs and MAGs. Gray dots indicate the average values. Genome completeness and contamination show all fecal MAG, fecal SAG, and oral SAG data. Metrics for high- or medium-quality genomes include quality (defined as completeness minus 5 × contamination), total length, contig count, CDS count, GC content, N50, tRNA repertoire, and rRNAs

The workflow and an overview of the bbsag20 dataset are shown in Fig. 1. Briefly, we performed single-cell genome sequencing [21] of saliva (total, 924,058,281,770 bp; mean, 75,310,373 bp/SAG) and feces (total, 1,302,352,706,360 bp; mean, 66,500,853 bp/SAG) collected from the Japanese participants (Supplementary Table 1). From 32 saliva samples, we obtained 11,809 bacterial SAGs, with an average of 369 SAGs (66 species) per sample. From 51 fecal samples, we obtained 19,042 bacterial SAGs, with an average of 373 SAGs (54 species) per sample. For the same set of 51 fecal samples, shotgun metagenome sequencing was conducted, yielding a total of 405,617,601,132 bp with a mean of 7,953,286,297 bp per metagenome. The salivary metagenomes did not reach sufficient quality due to the predominant human-derived DNA. The fecal metagenomes produced 1544 MAGs, averaging 30 MAGs per sample, as shown in Fig. 1a. According to standards by the Genomic Standards Consortium [25], 17,202 SAGs (55.76%) and 869 MAGs (56.28%) were classified as high- or medium-quality (Fig. 1a, b and Supplementary Table 2). When examining shared sequence information, SAG contigs shared, on average, a 49.5% overlap with metagenome assembly contigs, ranging between 31.9 and 84.0% (Supplementary Fig. 1). Conversely, the overlap with MAGs averaged 30.6%, ranging from 6.9 to 80.4%. Although the commonality of sequences obtained by metagenomics and single-cell genomics depends on the sample, more than half of the sequences were obtained in a method-dependent manner. These disparities underscore the unique genomic information yielded by single-cell genomics compared with metagenomics.

Comparisons of genome quality (Fig. 1b) showed that high- or medium-quality SAGs tended to have slightly lower quality (mean 61.5), higher contig counts (mean 364.2), and fewer tRNA genes than MAGs. A striking difference was observed in the recovery of rRNA genes, with MAG containing almost no rRNA (0.0069%), whereas 94.8% of fecal SAGs contained 16S rRNA genes, and 36.6% contained full-set rRNA genes (Fig. 1b). This lack of rRNA sequence challenge resulted in the production of a large number of semi-HQ MAGs, representing over a quarter of all MAGs, marked by the absence of rRNA genes, yet showing > 90% completeness and < 5% contamination. Participant-wise species distributions revealed 25–77 (mean 45) species in oral SAGs, 3–54 (mean 30) species in fecal SAGs, and 2–38 species (mean 17) in fecal MAGs. Notably, a Crohn’s disease patient had almost all SAGs (326 of 328) attributed to *Clostridium perfringens*, which causes gas gangrene and enterotoxemia.

**Fig. 2:**
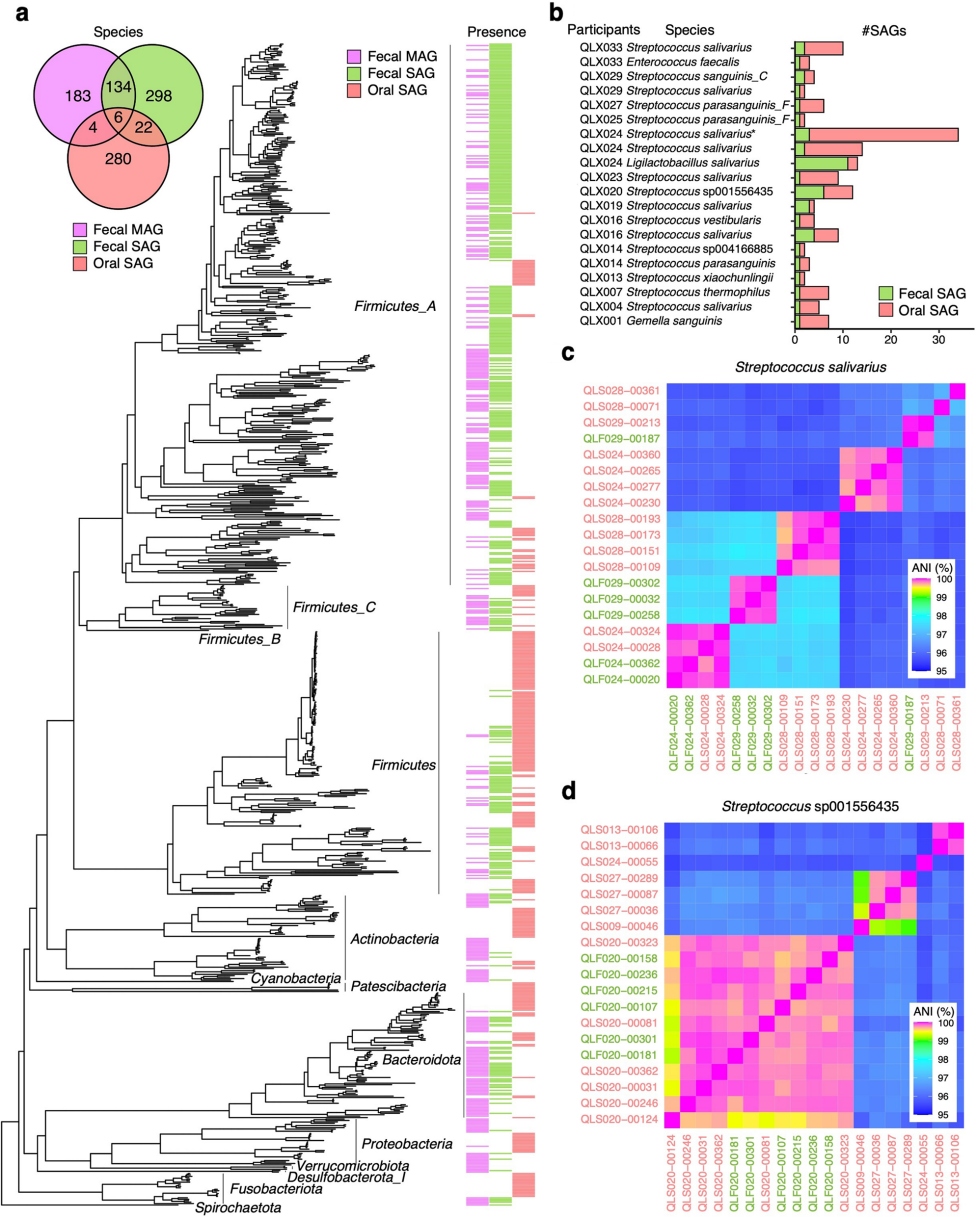
Taxonomy of bbsag20 for human oral and fecal bacteria. **a**, (left) Venn diagram visualizing the species found by fecal MAGs, fecal SAGs, and oral SAGs. (right) Phylogenetic tree representing 811 species obtained from all medium- or high-quality 17,202 SAGs and 869 MAGs. The colored strips show the presence of genomes in each method. **b** A list of the 12 species consistently present in both the oral and fecal microbiomes of the participants. The number of SAGs obtained is shown in different colors depend on samples. **c** ANI heatmap for *Streptococcus salivarius* across SAGs from saliva (salmon) and feces (green). **d** ANI heatmap for *Streptococcus* sp001556435 across SAGs from saliva (salmon) and feces (green)

A phylogenetic tree along 17,202 oral and fecal SAGs and 869 MAGs was retrieved through phyloT [26] and displayed varying taxonomic biases between SAGs and MAGs (Fig. 2a). A majority of SAGs identified deep genomic diversity across related species in specific lineages, whereas MAGs covered a broader range of lineages. In particular, 96.4% of SAGs (Fig. 2a; green strips) targeted 419 species of *Firmicutes*, currently renamed *Bacillota*, which are largely absent in MAGs. Of the 460 fecal SAG species identified, 320 were exclusive to SAGs, constituting 49.5% of the combined 647 species from fecal SAGs and MAGs (Fig. 2a and Supplementary Table 2). In contrast, MAGs identified 327 species (Fig. 2a; magenta strips), some of which (187 species) were uncharted in the SAG datasets. The predominance of *Firmicutes* (Gram-positive) in fecal SAGs was similar to that observed in our previous study [14]. These observations could result from inherent sample biases or potentially because certain species, such as Gram-negative bacteria, are susceptible to aerobic sample processing, solvent-induced lysis during sample preservation [24, 27], and freezing-induced stress, impeding their recovery through single-cell genome sequencing. Given that single-cell genomics can rectify the phylogenetic biases overlooked in metagenomics and provide strain genomes of closely related species, jointly leveraging both techniques promises a comprehensive genomic reference to unravel microbial diversity.

### Cell-resolved SAGs revealed oral-to-gut bacterial translocation

The oral microbiome comprises over 700 species and has been implicated in various systemic diseases [28], including afflictions of the central nervous system, gastrointestinal system, respiratory system, and hypertension [29]. While recent research suggests that 125 out of 310 oral species can be found in both the saliva and feces of 470 individuals across five countries, as determined by shotgun metagenome sequencing [30], there exists a contrasting study challenging the colonization of oral bacteria in the gut [31]. However, metagenomics might overstate the extent of oral bacterial translocation to the gut, especially because they also detect DNA fragments from lysed cells.

To investigate the translocation of oral bacteria to the gut at the cellular level, we analyzed the taxonomy of SAGs from both saliva (7136 SAGs across 312 species) and feces (10,066 SAGs across 460 species) for each participant. The overlap between these two microbiomes was limited, with only 12 species from four genera in oral SAG detected in fecal SAGs (Fig. 2b). These included *Streptococcus* (nine species), *Enterococcus* (one species), *Ligilatobacillus* (one species), and *Gemella* (one species). These bacterial candidates for translocation were identified based on oral and fecal SAG pairs that showed a Jaccard index > 0.21 in Dashing2 [32] within the same participants. While we found no relationships between these bacterial translocations and metadata of the participants (e.g., age, gender, or diseases), it is notable that the genus *Streptococcus* exhibited varying species detection trends across participants, and some participants even showed the translocation of multiple species. In total, 14 of the 32 participants, including four who were healthy, displayed signs of translocation.

For the validation of oral-to-gut bacterial translocation, we identified strains between fecal and oral SAGs according to average nucleotide identity (ANI) comparisons. Figures 2c and d show ANIs for *Streptococcus salivarius* and *Streptococcus* sp001556435 SAGs derived from fecal and oral samples. In Fig. 2c for *S. salivarius*, the three fecal SAGs (QLF024-00020, QLF024-00362, and QLF029-00187) showed high ANI of 99.5–100% with oral SAGs from the same participant while showing 95–97% ANI with the SAGs from other participants or other strain SAGs from the same participant. In Fig. 2d for *Streptococcus* sp001556435, six fecal SAGs in QLF020 showed high ANI of 99.6–100% with oral SAGs from the same participant while showing 95–97% ANI with the SAGs from other participants.

Our data present initial evidence of bacterial translocation from the oral cavity to the gut based on cell-resolved SAG identity. Although further validation is needed to evaluate the existence of biological systems that allow oral bacteria to survive in the gastric environment or colonize the fecal microbiota, the strain identity between oral and fecal SAGs observed in this study proves the presence of oral bacteria that have tolerated harsh environmental changes. Utilizing cell-resolved SAGs may be instrumental for culture-independent evaluations of bacterial viability and colonization, especially when exploring the interactions between distinct bacterial species across environments.

### Linking mobilome and resistome in the human-associated microbiome

MGEs, such as plasmids and phages, are transferred across bacterial hosts and sometimes act as carriers of ARGs, thereby conferring antimicrobial resistance to bacteria [33, 34]. Despite efforts in culturomics [35, 36] and metagenomics, which have accumulated hundreds of thousands of MGEs [37, 38], current genomic analyses have found it challenging to reveal the prevalence of MGEs in individual bacteria. Unlike traditional methods, SAGs can directly determine the host and MGE relationships based on single-cell-resolved information. To integrate the mobilome and resistome information from SAGs, we detected plasmids using Platon [39], which matched known databases, and identified phages using PhageBoost [40]. The phages were of complete, high-, or medium-quality and contained viral genes [41] obtained from both SAGs and MAGs. From the oral SAGs, we identified 1491 plasmid sequences and 5827 phage sequences (Fig. 1a and Supplementary Tables 3 and 4). In fecal SAGs, we identified 5087 plasmid and 4226 phage sequences, respectively. Oral SAGs tend to have fewer plasmids than fecal SAGs, with 0.21 plasmids/genome compared to 0.51 plasmids/genome. In contrast, oral SAGs contained more phages, with 0.82 phages/genome compared to 0.42 phages/genome. In contrast, of the 2227 plasmids and 633 phages identified in fecal metagenomes, only 10.78% and 33.65%, respectively, were binned into MAGs, highlighting the challenge of associating MGEs with MAGs. Participant-wise plasmid distributions revealed 2–521 in oral SAG, 4–1331 in fecal SAG, 0–34 in fecal MAG, and 4–130 in fecal MG (Supplementary Fig. 2). Participant-wise phage distributions revealed 32–471 in oral SAG, 11–191 in fecal SAG, 0–16 in fecal MAG, and 1–23 in fecal MG samples. The majority (83.1–96.7%) of the oral and fecal phages found were *Caudoviricetes*, with complete, high-, or medium-quality viral genomes acquired in thousands (Supplementary Table 4).

**Fig. 3:**
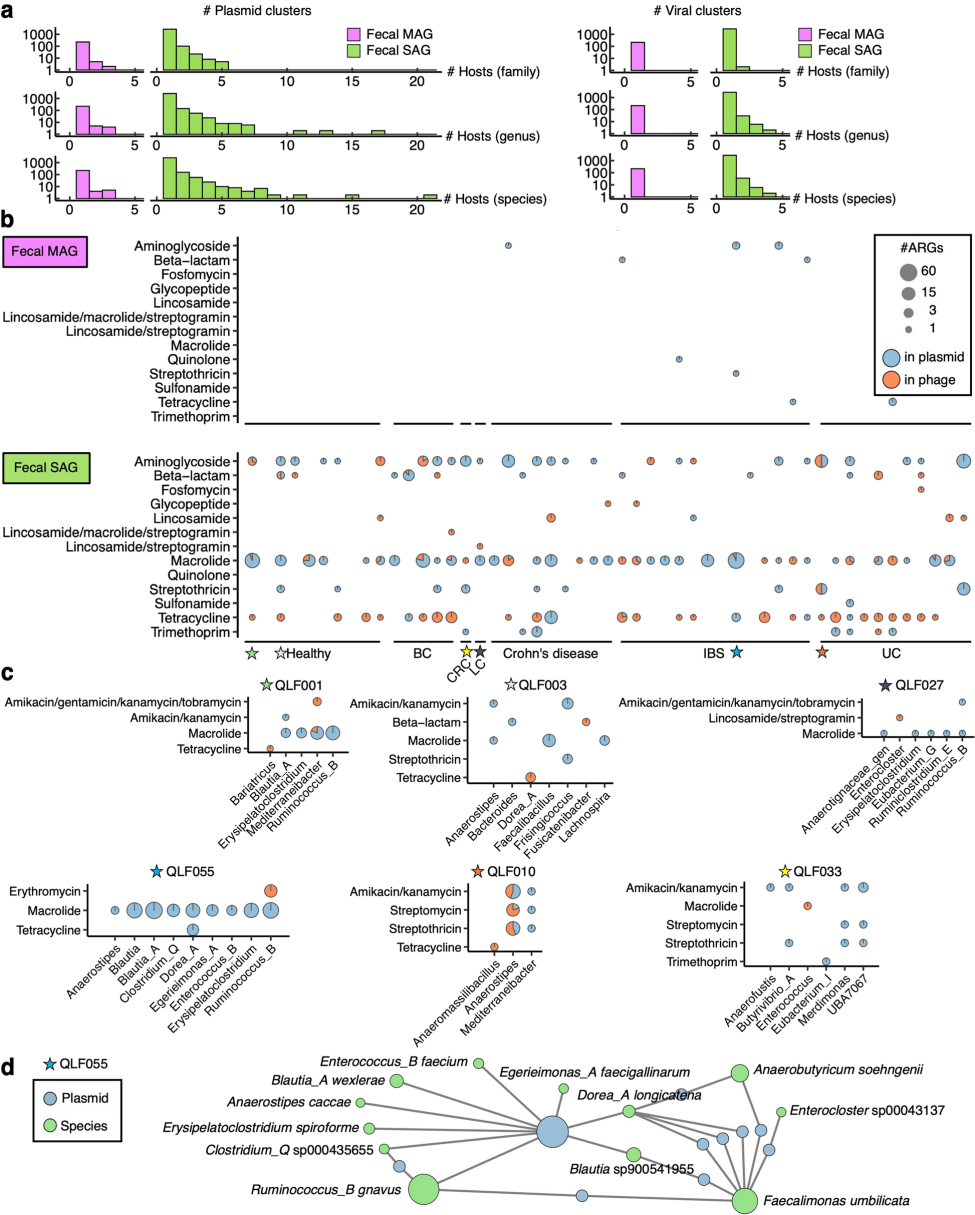
Detailed examination of mobilomes and resistomes in human-associated microbiomes at single-cell resolution. **a** Determination of the host spectrum of plasmids and phages. To avoid redundant counts, similar plasmids or phage sequences were grouped into clusters. The predicted host numbers are depicted in histograms, distinguishing between SAGs and MAGs across different taxonomic ranks. **b** Distribution of ARGs in MGEs. ARG (class) presence and genetic context are visualized as pie charts. The x-axis labels detail the medical condition associated with each sample (Healthy; BC, breast cancer; CRC, colorectal cancer; LC, lung cancer; IBS, irritable bowel syndrome; UC, ulcerative colitis). **c** Comparison of ARGs (subclass) in MGEs among participants. Six resistomes in the gut microbiome (QLF001, QLF003, QLF010, QLF027, QLF033, and QLF055, marked with stars in **c**) are presented. **d** A network diagram depicted the links between the plasmid and its host genome at the species level in QLF055. Lines represent the connections between bacterial hosts and plasmids

Next, we evaluated the number of bacterial host lineages and assumed host ranges for each MGE. Both plasmid and phage contigs were deduplicated by clustering using MMseqs2 [42] at 90% similarity and coverage, and the number of identical clusters observed was recorded according to taxonomic categories. Both plasmids and phages showed distinct broad host ranges when comparing SAGs with MAGs. The histogram showed that 21 species for plasmids and four species for phages were the maximal MGE host ranges observed in fecal SAGs, but only three species for plasmids and one species for phages were observed in fecal MAGs (Fig. 3a). These observations in SAGs are consistent with a recent study of broad-host-range plasmids using thousands of isolated genomes in public databases [43] and demonstrate the advantage of single-cell genomics for determining the bacterial host ranges of MGEs, which are often underestimated using conventional metagenomic approaches.

We identified 10,241 and 19,660 ARGs in oral and fecal SAGs, respectively (Supplementary Table 5), using AMRFinderPlus [44]. Metagenome assemblies displayed 2881 ARGs, with only 640 allocated to MAGs (Fig. 1a). Notably, fecal SAGs exhibited a higher count of ARGs than MAGs, with 1.95 ARGs/SAG and 0.74 ARGs/MAG. The repertoire of ARGs differed among the oral SAGs, fecal SAGs, metagenome assemblies, and MAGs. The efflux pump genes corresponding to fluoroquinolone resistance were exclusively found in oral SAGs (1329 of *pmrA* genes) but not in fecal SAGs or metagenomes (Supplementary Fig. 3 and Supplementary Table 5; 0 genes and 1 *qnrS1* gene, respectively). Conversely, fecal SAGs contained 1869 genes along with 26 aminoglycoside resistance genes, whereas oral SAGs had only 10 genes (Supplementary Fig. 3 and Supplementary Table 5). Regarding the disparities between SAGs and MAGs, 631 genes linked to trimethoprim resistance (*dfrA1*, *dfrA17*, *dfrF*, and *dfrG*) were found in fecal SAGs, while the metagenome and MAG had 21 and 1 genes, respectively. Metagenomes and MAGs showed distinct profiles in tetracycline resistance genes; only 29 genes were found in MAGs, despite 251 genes being found in metagenome assemblies, suggesting difficulty in binning ARGs to MAGs.

Understanding the mode of ARG transfer between bacteria is important for determining the emergence of drug-resistant bacteria. We integrated the mobilome and resistome of fecal SAGs and MAGs to determine the potential for ARG transfer associated with plasmids and phages (Fig. 3b). Importantly, only 2.8% (550/19,660) and 1.8% (12/640) of ARGs in fecal SAGs and MAGs were located on plasmids or phages, respectively, and the rest were found in the chromosome or unidentified (Supplementary Fig. 4). There was no obvious dependence of the resistome profiles on the participant background. MAGs detect a minimal number of MGE and ARG relationships, rendering sample comparisons challenging. In contrast, SAGs provided comprehensive data, revealing largely consistent mobilome and resistome profiles across samples (Fig. 3b). This provides an insight into the preferences of the transfer modes for each resistance. For instance, tetracycline resistance genes were mainly found in phages rather than plasmids, whereas those for macrolides were found in plasmids. Intriguingly, although the pattern of resistome possession in each individual was similar, each ARG was shown to be capable of being transferred via plasmids or phages.

Figure 3c shows the ARG subclasses and their bacterial hosts for the six participants. These bacterial host-specific ARG-MGE profiles suggest that the same resistance genes for macrolide (*ermB*) are transmitted via different modes to different bacterial taxa (Fig. 3c; QLF001, 003, 027, and 055). The distribution of resistance genes offers insight into their transmission patterns. For instance, resistance genes for amikacin/kanamycin (*aph(3′)-IIIa*) were predominantly found on plasmids in QLF033 SAGs. In QLF010 SAGs, nearly half of these genes were present in phages of bacterial genera that were not found in QLF033. In other cases, while QLF003 SAGs had *ermB* genes across plasmids from three genera, including *Anaerostipes*, *Faecalibacillus*, and *Lachnospira*, QLF055 SAGs had these genes in plasmids across nine genera, including *Anaerostipes*, *Blautia*, and others (Fig. 3c). The total number of plasmids detected from the nine genera of QLF055 SAGs was 52, which showed 100% sequence homology to each other, except for small gaps at the contig ends (Supplementary Fig. 5). Platon suggested a 99.94% identity between these partial contigs and NC_017962.1 (*Enterococcus faecium* DO plasmid 2), suggesting that macrolide resistance genes were potentially transferred via these plasmids to multiple gut bacterial species in the same participant (QLF055) as shown in a network diagram (Fig. 3d; center). Single-cell genomics represents a breakthrough in our ability to unveil intricate networks of mobilomes and resistomes on a per-sample basis (Supplementary Fig. 6). This information surpasses conventional metagenomics, highlighting dynamic gene exchanges through MGEs in the microbial landscapes of human hosts.

## Discussion

Our study introduced the bbsag20 dataset, which is a comprehensive collection of 17,202 SAGs and 869 MAGs from human saliva and feces. The qualitative similarities between SAGs and MAGs are notable, but the enhanced rRNA gene recovery in SAGs underscores their potential superiority in reference genomes for conventional analyses, including 16S rRNA amplicon sequencing. Both methods exhibited taxonomic biases, emphasizing the benefits of combining single-cell genomics and metagenomics to achieve a full species diversity snapshot. We noted pronounced taxonomic differences between oral and fecal SAGs, with a limited overlap of only 12 species. This mirrors earlier research highlighting the separate microbial niches in the oral cavity and gut, underlining the need for targeted sampling in microbiome studies.

In culture-free microbial research, single-cell genomics has emerged as a potent tool for addressing and filling the lacunae left by traditional metagenomic approaches. This assertion is bolstered by our findings, which highlight the superior sensitivity and precision of single-cell genomics, especially in the profiling of MGEs and ARGs. While some cutting-edge research aims to connect MAGs, MGEs, and ARGs using Hi-C metagenomics, the extensive sequence reads required often limit MGE and ARG detection [45–49]. Single-cell genomics, with its ability to overcome such challenges, offers a refined view of the complex dynamics among ARGs, MGEs, and their hosts.

From a public health perspective [50, 51], profiling of the microbiome, mobilome, and resistome highlights pathways to address growing concerns regarding antimicrobial resistance. Recognizing the spread of antimicrobial resistance, it is vital to understand the reservoirs and the transmission of ARGs [3, 46, 47, 52]. This knowledge will drive the development of strategies to prevent the spread of resistant pathogens. For example, discerning that specific resistance genes are mainly present in plasmids within certain bacterial groups may inform both monitoring and targeted interventions.

The proposed research approach has implications not only for health care but also for the environmental and agricultural sectors. With the spread of antimicrobial resistance through diverse ecosystems such as hospitals, farms, and water sources, a thorough understanding of ARG dynamics is essential for a comprehensive approach. Single-cell genomics has the potential to be a key tool for tracking genetic shifts across environments, enabling proactive measures and data-driven decision-making.

## Conclusions

Our study emphasizes the game-changing capacity of single-cell genomics in microbiome studies. This provides a new perspective on microbial communities, MGEs, and antimicrobial resistance patterns, and offers a renewed understanding of microbial interplay. The bbsag20 dataset demonstrates the effectiveness of this method. Our data highlight the potential of single-cell genomics for monitoring the dynamics of MGEs and ARGs in the microbiome across people, animals, and the environment.

## Methods

### Experimental design and sample collection

All human subjects signed a written informed consent form, and the project was approved by the ethics review committee at Yamauchi Clinic (No. 2020–08-00092). All methods were conducted in accordance with the guidelines and regulations outlined by the ethics approval. Preserved feces were collected in 15 mL vials containing 3 mL GuSCN solution (FS-0002; TechnoSuruga Laboratory Co., Ltd., Shizuoka, Japan) and stored at 4 °C for a maximum of 2 weeks prior to single-cell encapsulation in droplets or DNA extraction. Preserved saliva was collected in OMNIgene ORAL (OM-501; KYODO INTERNATIONAL INC., Kanagawa, Japan) and stored at 4 °C for a maximum of two weeks prior to single-cell encapsulation in droplets or DNA extraction.

### Single-cell genome sequencing

Following the suspension of human feces in the GuSCN solution (500 μL), the supernatant was recovered by centrifugation at 2000 × *g* for 30 s, followed by filtration through a 35-μm nylon mesh and centrifugation at 8000 × *g* for 5 min. The resulting cell pellets were suspended in DPBS and centrifuged twice at 8000 × *g* for 5 min. Bacterial cell suspensions were prepared in 100–500 μL of PBS and used in the following steps.

Single-cell genome amplification was performed using the SAG-gel platform, as described in our previous reports [21, 22]. Prior to single-cell encapsulation, cell suspensions were adjusted to 0.3–0.4 cells/droplets in 1.5% agarose in DPBS to prevent encapsulation of multiple cells in single droplets. Using an On-chip Droplet Generator (On-chip Biotechnologies Co., Ltd., Tokyo, Japan), single bacterial cells were encapsulated in droplets and collected in a 1.5 mL tube, which was chilled on ice for 15 min to form the gel matrix. Following solidification, the collected droplets were broken using 1H, 1H, 2H, 2H-perfluoro-1-octanol (Sigma-Aldrich, STL, MO, USA) to collect the capsules. The gel capsules were washed with 500 μL of acetone (FUJIFILM Wako Pure Chemical Corporation, Osaka, Japan), and the solution was mixed vigorously and centrifuged. The acetone supernatant was removed, 500 μL of isopropanol (FUJIFILM Wako Pure Chemical Corporation) was added, and the solution was mixed vigorously and centrifuged. The isopropanol supernatant was removed, and the gel capsules were washed three times with 500 μL of DPBS. Individual cells in capsules were then lysed by submerging the gel capsules in lysis solutions: first, 50 U/μL Ready-Lyse Lysozyme Solution (Lucigen, WI, USA); 2 U/mL Zymolyase (Zymo Research Corporation, CA, USA); 22 U/mL lysostaphin (Sigma-Aldrich); and 250 U/mL mutanolysin (Sigma-Aldrich) in DPBS at 37 °C overnight; second, 0.5 mg/mL achromopeptidase (FUJIFILM Wako Pure Chemical Corporation) in PBS at 37 °C for 6–8 h; and third, 1 mg/mL Proteinase K (Promega Corporation, WI, USA) with 0.5% SDS in PBS at 40 °C overnight. At each reagent replacement step, the gel capsules were washed three times with DPBS and subsequently resuspended in the next solution.

Following lysis, the gel capsules were washed five times with DPBS, and the supernatant was removed. The capsules were then suspended in Buffer D2 and subjected to multiple displacement amplification (MDA) using REPLI-g Single Cell Kit (QIAGEN, Germany). Following MDA treatment at 30 °C for 3 h, the gel capsules were washed three times with 500 μL of DPBS. Thereafter, the capsules were stained with 1 × SYBR Green I (Thermo Fisher Scientific, MA, USA) in DPBS and observed with fluorescence microscopy BZ-X810 (KEYENCE CORPORATION, Osaka, Japan) to count the number of fluorescence-positive gel capsules. Following confirmation of DNA amplification based on the presence of green fluorescence in the gel, fluorescence-positive capsules were sorted into 384-well plates using a BD FACSMelody cell sorter (BD Biosciences, Tokyo, Japan) equipped with a 488-nm excitation laser.

Following droplet sorting, 384-well plates were subjected to the second round of MDA or were stored at − 30 °C. Following gel capsule collection in 384-well plates, second-round MDA treatment was performed using the REPLI-g Single Cell Kit. Buffer D2 was added to each well and incubated at 65 °C for 10 min. Thereafter, the MDA mixture was added and incubated at 30 °C for 120 min. The MDA reaction was terminated by heating at 65 °C for 3 min.

For sequencing analysis, sequencing SAG libraries were prepared from the second-round MDA product using the QIAseq FX DNA Library Kit (QIAGEN). Aliquots of SAGs were transferred to replica plates for DNA yield quantification using Quant-iT dsDNA Broad-Range (BR) Assay Kit (Thermo Fisher Scientific) prior to library preparation. Ligation adaptors were modified using TruSeq-Compatible Full-length Adapters UDI (Integrated DNA Technologies, Inc., IW, USA). Each SAG library was sequenced using an Illumina HiSeq X Ten System with a 2 × 150 bp configuration at Macrogen Japan Corp. (Tokyo, Japan) or using an Illumina NextSeq 2000 System with a 2 × 150 bp configuration.

### Shotgun metagenome sequencing

The QIAamp PowerFecal Pro DNA Kit (QIAGEN) was used for total DNA extraction from the saliva and fecal samples. Metagenomic sequencing libraries were constructed from extracted DNA samples with 10 μL (1/5 volume) reactions using the QIAseq FX DNA Library Kit (QIAGEN). Each metagenomic sequencing library was sequenced using the Illumina NextSeq 2000 System 2 × 150 bp configuration.

### Genome analysis

Adapter sequences and low-quality reads were eliminated from raw sequence reads of metagenome sequences and single-cell genome sequences using bbduk.sh (version 38.90; https://sourceforge.net/projects/bbmap/) with following options (qtrim=r trimq=10 minlength=40 maxns=1 minavgquality=15). These quality-controlled reads of single-cell genomes were assembled de novo into contigs using SPAdes (v3.14.0) [53] with the following options (--sc --careful --disable-rr --disable-gzip-output). Contigs shorter than 1000 bp were excluded from the SAG assemblies. Metagenome reads were assembled using SPAdes with the following options (--meta). MAGs were constructed using three binning tools, including CONCOCT (v1.0.0) [54], MaxBin 2 (v2.2.6) [55], and MetaBAT 2 (v2.12.1) [56], with default options, and DAS_Tool (v1.1.2) [57] was used to refine the binning results. CDSs, rRNAs, and tRNAs were predicted from the SAGs and MAGs using Prokka (v1.14.6) [58] with the following options (--rawproduct). The completeness and contamination of SAGs and MAGs were evaluated using CheckM (v1.1.2) [59] lineage workflow with default options. Taxonomy identification was performed using GTDB-Tk (v2.1.0) [60] with default options, and GTDB release 207.

### Alignment of metagenome assemblies and single-cell genome assemblies

The contig overlap lengths between metagenome assemblies and SAGs were calculated based on the results of BLASTn with the following options (-outfmt 6 -num_threads 4 -perc_identity 95 -max_target_seqs 50,000). Only hits above 1000 bp and 99% similarity were extracted using the awk command (awk ‘{if($3 >= 99 && $4 >= 1000) print $0}’). The redundancy was removed by piling up the overlap hits using awk and BEDTools [61] (cut -f 2,9,10 input.tsv | sort | uniq | awk ‘{if($2 > $3) print $1 "\t" $3–1 "\t" $2 "\t." "\t0" "\t + "; else if($2 < $3) print $1 "\t" $2–1 "\t" $3 "\t." "\t0" "\t + ";}’ | sort -k1,1 V -k2,2 V -k3,3 V | uniq | bedtools merge -i - | awk ‘BEGIN{OFS = "\t"}{$4 = $3-$2; print $0}’ | sed "1i contig\tstart\tend\tlength").

### Phylogenetic analysis of oral and fecal bacterial genomes

A total of 7136 oral SAGs, 10,066 fecal SAGs, and 869 fecal MAGs above medium-quality were retrieved from the bbsag20 dataset. The undetermined taxa in GTDB-Tk (release 207) were removed and 811 unique taxa were used in the following analysis. The phylogenetic tree was retrieved using phyloT with the removal of one species (*Methanobrevibacter_A smithii*) due to an error in phyloT. Tree visualization and annotation were performed using an R package “ggtree” [62].

### Identification of plasmid, phage, and ARGs

SAGs (oral, 7136; feces: 10,066); fecal metagenome assemblies (*n* = 51); and MAGs (*n* = 869) above medium quality were used for mobilome and resistome analysis. Plasmids were predicted using Platon (version 1.6) [39] with default parameters (platon --db ${platondb} --output ${sampleid} --verbose --threads ${cpus} ${fna}). The list was filtered with “#Plasmid Hits” = 1 (True). Phages were predicted using PhageBoost (version 0.1.7) [40] with default parameters (PhageBoost -f ${fna} -o ${sampleid} --threads ${cpus}), and their quality were assessed using CheckV (v1.0.1) [41] with following command (checkv end_to_end -d checkv-db-v1.4 -t 4 ${input}.fna result/checkv/${input}). Only hits with checkv_quality = Medium-quality, High-quality, or Complete having at least one viral gene (viral_genes > = 1) were used in the following analysis. We employed clustering on the plasmids and phages by MMseqs2 (version 13.45111) [42] using a cut-off threshold both of above 90% coverage and similarity (mmseqs cluster --threads ${cpu} --cov-mode 0 -c 0.90 --min-seq-id 0.90 ${mmseqs_db} ${cluster_db} ${cluster_db_tmp}; mmseqs createtsv --threads ${cpu} ${mmseqs_db} ${mmseqs_db} ${cluster_db} ${sampleid}_c90s90.tsv). ARGs were identified using the NCBI AMRFinderPlus [44] with following options (amrfinder --plus -p ${faa} -n ${fna} -g ${gff} --threads ${cpus} -a prokka -o ${sampleid}.tsv --nucleotide_output ${sampleid}_amrfp.fna --protein_output ${sampleid}_amrfp.faa). To exclude virulence genes, heavy metal resistance genes, and partial genes, we removed hits with Method = PARTIALP, PARTIAL_CONTIG_ENDX, PARTIAL_CONTIG_ENDP, PARTIALX, INTERNAL_STOP) and used only hits with Element type = AMR.

### Estimation of host ranges of plasmids and phages

The filtered plasmids and phages data were combined with metadata of high- or medium-quality SAGs or MAGs containing genome ID, sample ID, and GTDB taxonomy (release 207) based on their contig ID. We counted the unique taxa after grouping them by family, genus, or species for each plasmid or phage cluster using the R program. The frequencies of the unique taxa were plotted.

### Visualization of mobilome and resistome in individual participants

The identified plasmids and ARGs were combined based on contig ID. ARGs in phage genomic regions were extracted using bedtools (sed 1d ${amrfp}.tsv | awk ‘BEGIN {OFS = "\t"}{print $2 "\t" $3–1 "\t" $4 "\tAMRFinderPlus\t.\t" $5}’ > ${sampleid}_amrfp.bed; sed 1,2d ${sampleid}_phageboost.gff | sed "s/gnl|bB|//g" | sed "s/QLF…/\1sag/g" | awk ‘BEGIN {OFS = "\t"}{print $1 "\t" $4–1 "\t" $5 "\tPhageBoost\t.\t + "}’ > ${sampleid}_phageboost.bed; bedtools intersect -a ${sampleid}_amrfp.bed -b ${sampleid}_phageboost.bed -f 1.00 -wa). The numbers of ARGs in plasmids or phages for each ARG class were counted by sample ID and plotted using the R package “scatterpie.” The number of ARGs in plasmids or phages for each ARG subclass was counted by genus and plotted depending on the sample ID. The network diagram between plasmids and ARGs was generated using the R package “igraph” [63] and visualized using Gephi [64].

## Supplementary Information

Supplementary Material 1: Supplementary Figs. 1–6.

Supplementary Material 2: Supplementary Table 1. Participant information for this study and statistics of raw reads deposited in SRA.

Supplementary Material 3: Supplementary Table 2. List of SAGs and MAGs derived from the study, including metrics such as genomic cluster ID determined by Dashing2, genome ID, completeness, contamination, quality score, contig count, total genomic length, N50, GC content (%), CDS count, type, sample ID, sample source, and taxonomy based on the GTDB (release 207), counts of tRNAs, rRNAs, plasmids, phages, and ARGs. Species summary for oral SAGs, fecal SAGs, and MAGs are also included.

Supplementary Material 4: Supplementary Table 3. List of plasmids identified in metagenomes, MAGs, and SAGs using Platon.

Supplementary Material 5: Supplementary Table 4. List of phages detected in metagenomes, MAGs, and SAGs using PhageBoost, further refined by CheckV and classified by geNomad.

Supplementary Material 6: Supplementary Table 5. List of ARGs detected in metagenomes, MAGs, and SAGs using AMRFinderPlus.

## Data Availability

The raw data produced in this study were deposited at NCBI under BioProject ID PRJNA1030952. The genome assemblies and annotation data produced in this study were deposited at FigShare + (doi: 10.25452/figshare.plus.24473008.v2).
