# Supplementary material for: A single amplified genome catalog reveals the dynamics of mobilome and resistome in the human microbiome
Source: Microbiome. 2024 Oct 2;12:188. doi: 10.1186/s40168-024-01903-z (PMC11446047; doi:10.1186/s40168-024-01903-z)
Supplement: Supplementary file 2 — Supplementary Material 1: Supplementary Figs. 1–6. [file 40168_2024_1903_MOESM1_ESM.pptx]

## Slide 1
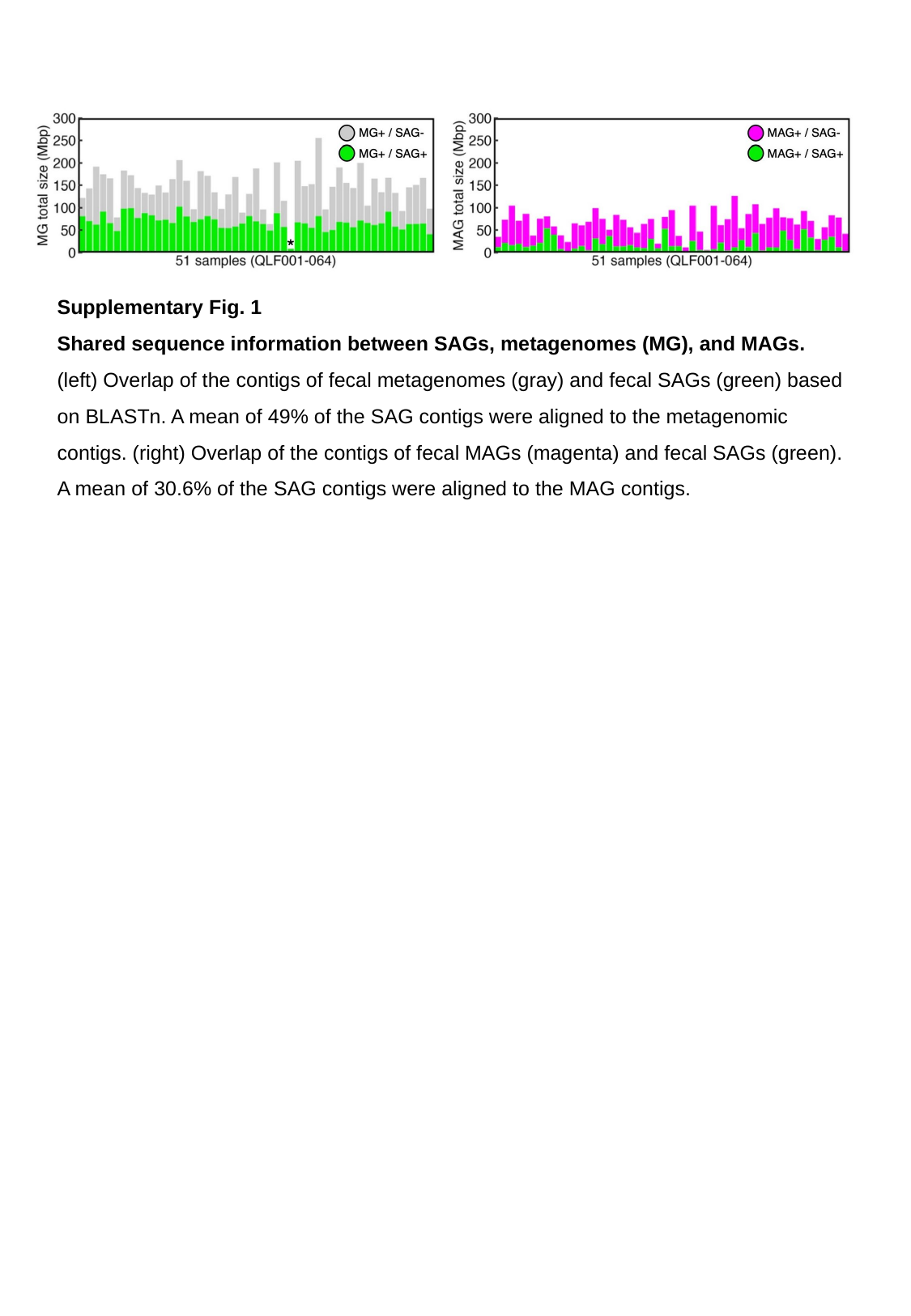

Supplementary Fig. 1
Shared sequence information between SAGs, metagenomes (MG), and MAGs.
(left) Overlap of the contigs of fecal metagenomes (gray) and fecal SAGs (green) based on BLASTn. A mean of 49% of the SAG contigs were aligned to the metagenomic contigs. (right) Overlap of the contigs of fecal MAGs (magenta) and fecal SAGs (green). A mean of 30.6% of the SAG contigs were aligned to the MAG contigs.

## Slide 2
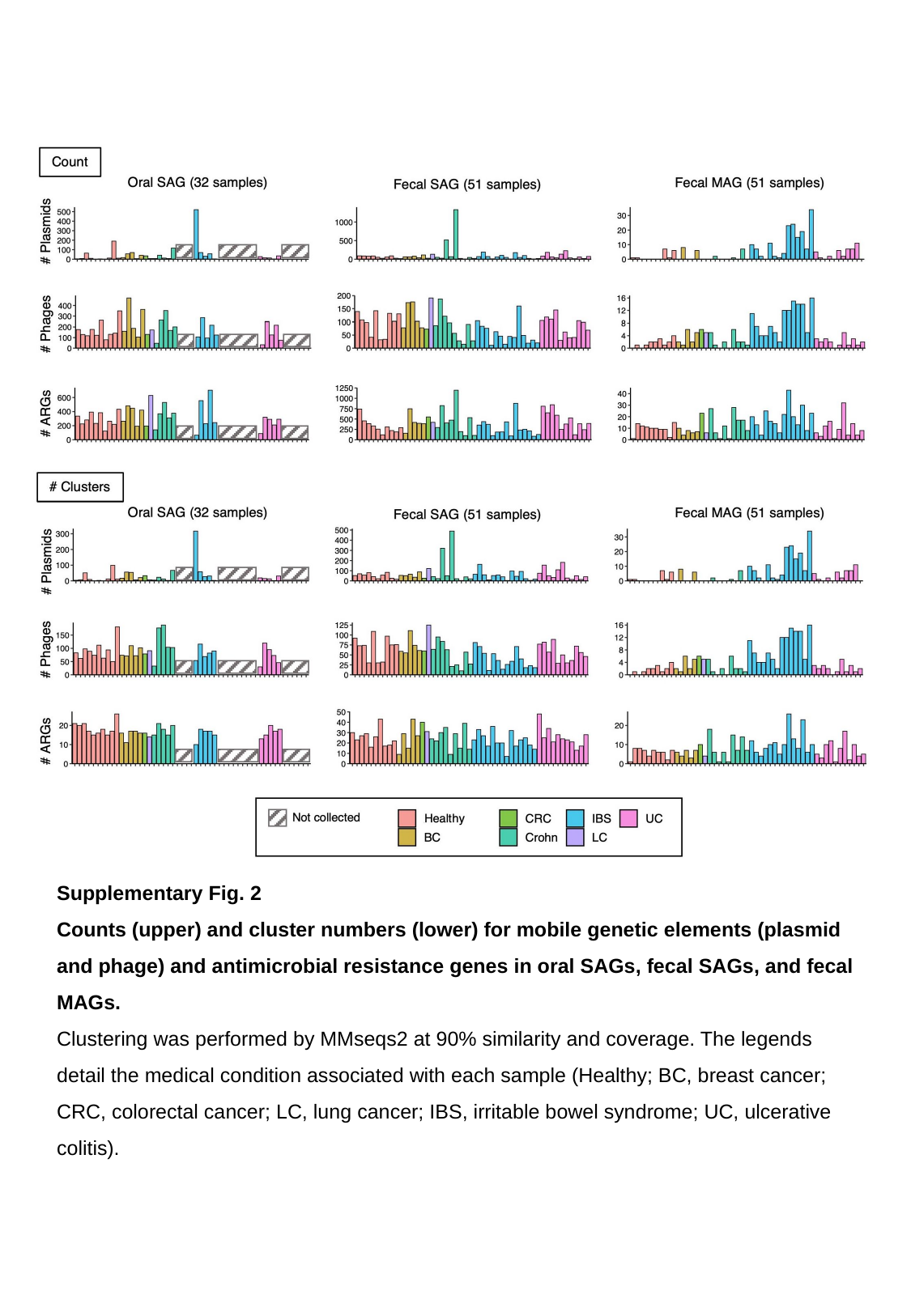

Supplementary Fig. 2
Counts (upper) and cluster numbers (lower) for mobile genetic elements (plasmid and phage) and antimicrobial resistance genes in oral SAGs, fecal SAGs, and fecal MAGs.
Clustering was performed by MMseqs2 at 90% similarity and coverage. The legends detail the medical condition associated with each sample (Healthy; BC, breast cancer; CRC, colorectal cancer; LC, lung cancer; IBS, irritable bowel syndrome; UC, ulcerative colitis).

## Slide 3
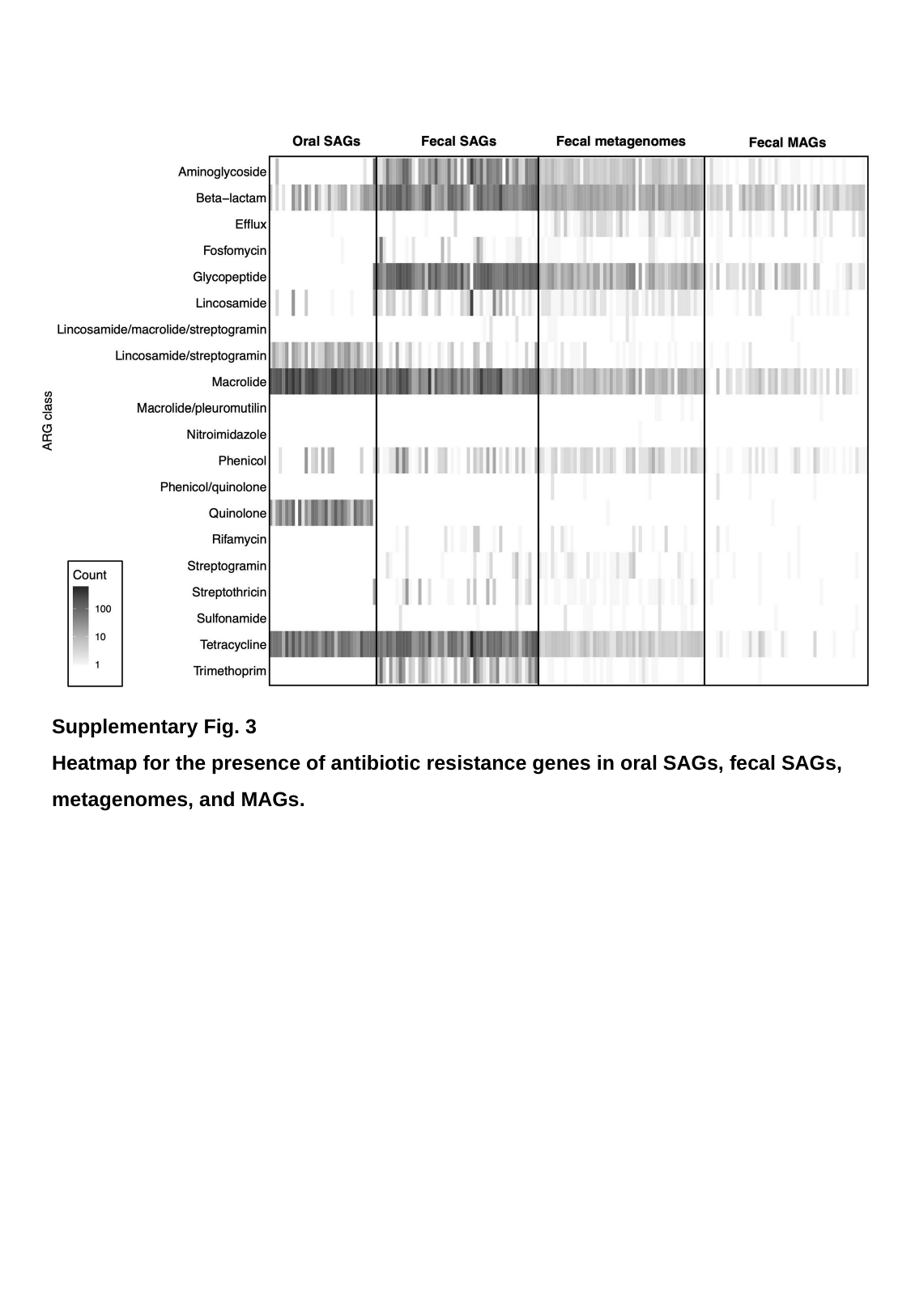

Supplementary Fig. 3
Heatmap for the presence of antibiotic resistance genes in oral SAGs, fecal SAGs, metagenomes, and MAGs.

## Slide 4
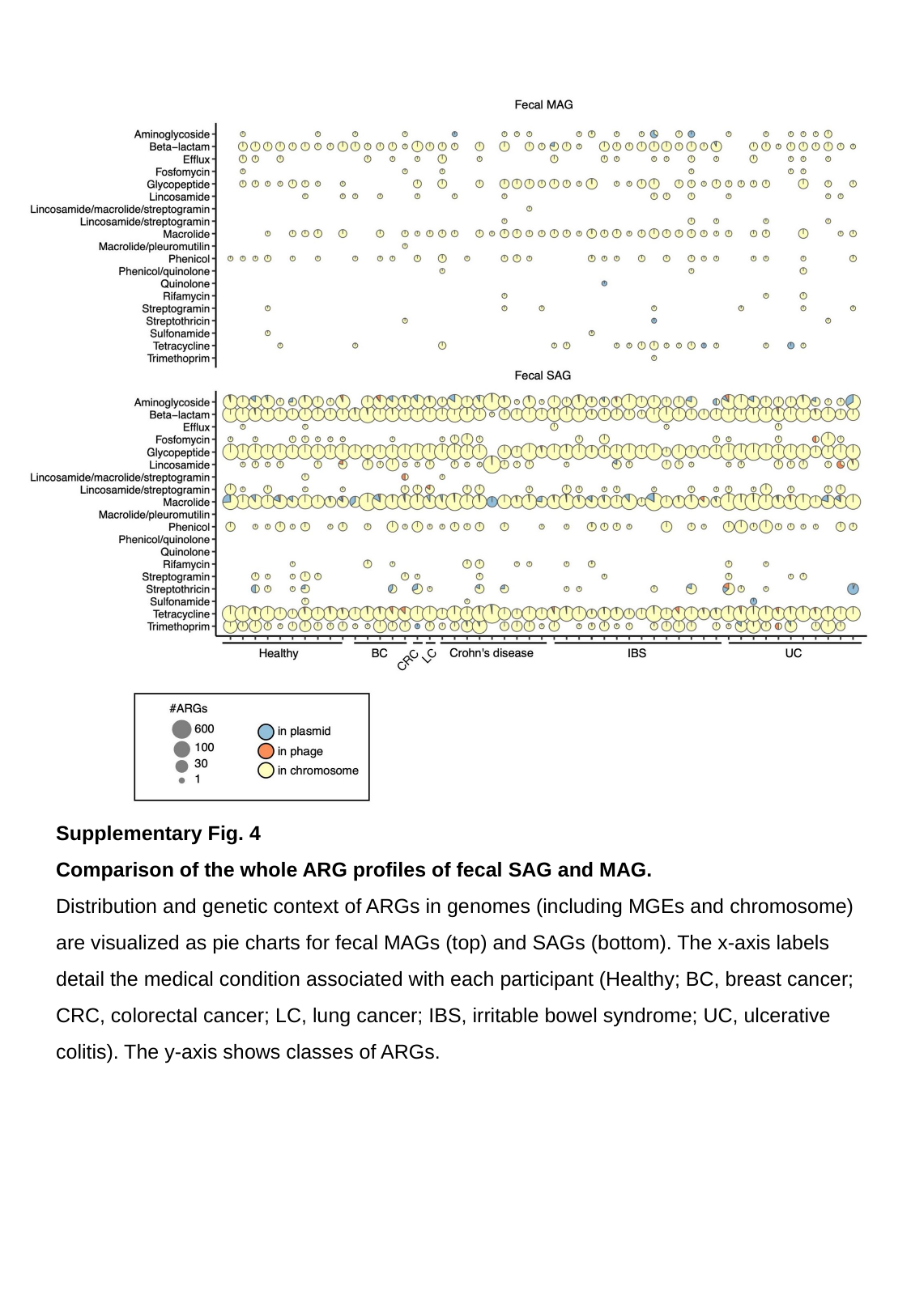

Supplementary Fig. 4
Comparison of the whole ARG profiles of fecal SAG and MAG.
Distribution and genetic context of ARGs in genomes (including MGEs and chromosome) are visualized as pie charts for fecal MAGs (top) and SAGs (bottom). The x-axis labels detail the medical condition associated with each participant (Healthy; BC, breast cancer; CRC, colorectal cancer; LC, lung cancer; IBS, irritable bowel syndrome; UC, ulcerative colitis). The y-axis shows classes of ARGs.

## Slide 5
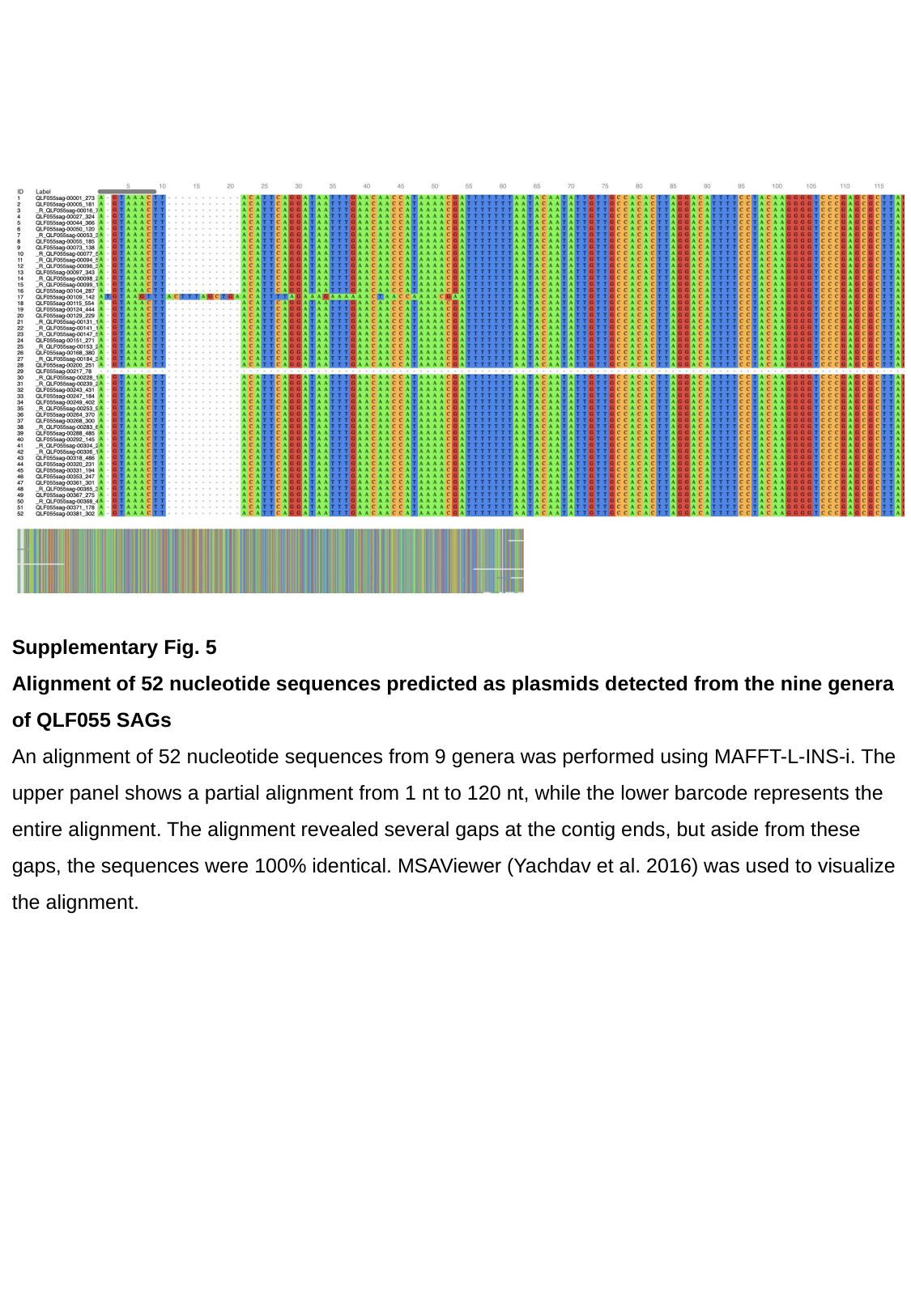

Supplementary Fig. 5
Alignment of 52 nucleotide sequences predicted as plasmids detected from the nine genera of QLF055 SAGs
An alignment of 52 nucleotide sequences from 9 genera was performed using MAFFT-L-INS-i. The upper panel shows a partial alignment from 1 nt to 120 nt, while the lower barcode represents the entire alignment. The alignment revealed several gaps at the contig ends, but aside from these gaps, the sequences were 100% identical. MSAViewer (Yachdav et al. 2016) was used to visualize the alignment.

## Slide 6
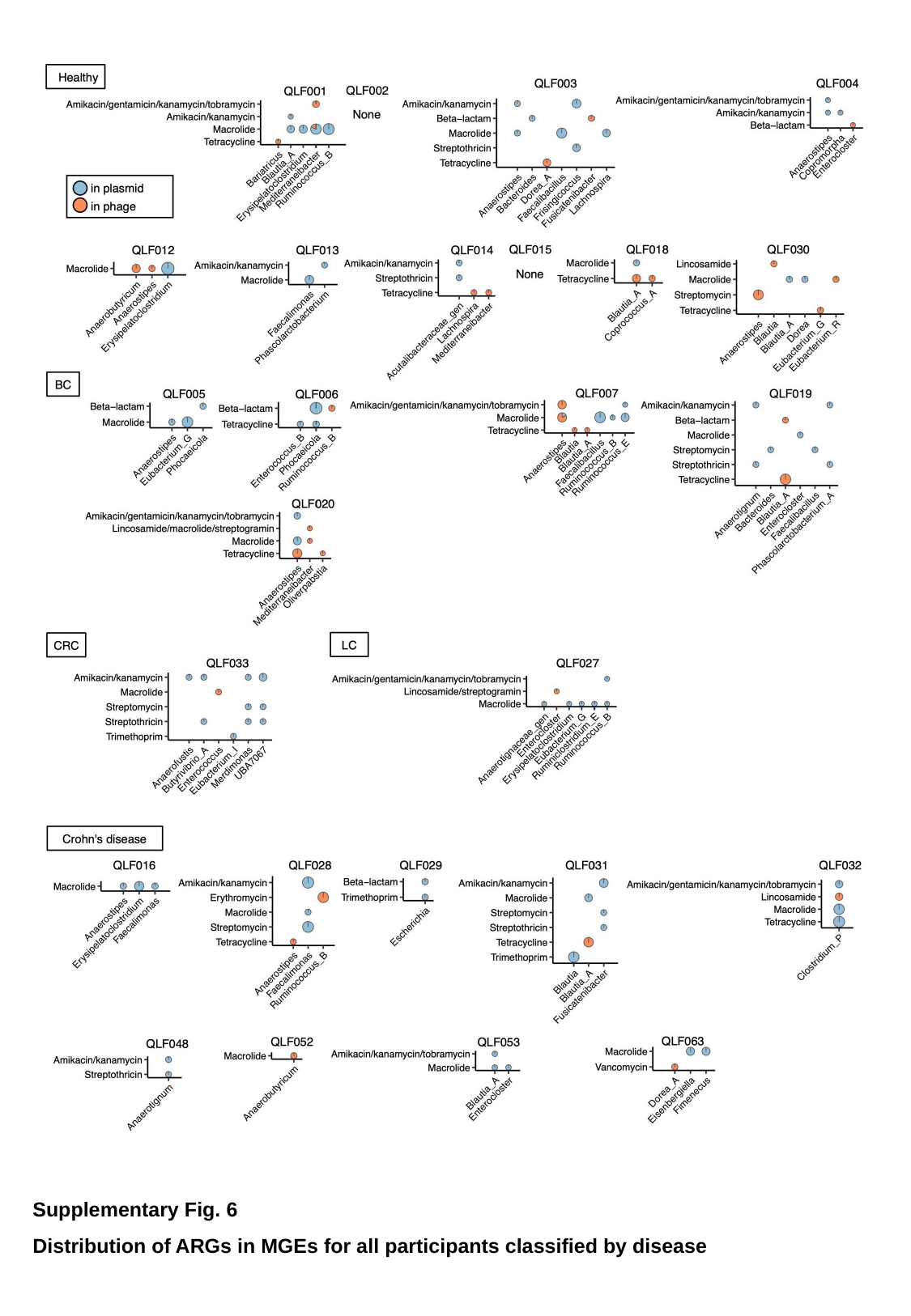

Supplementary Fig. 6
Distribution of ARGs in MGEs for all participants classified by disease

## Slide 7
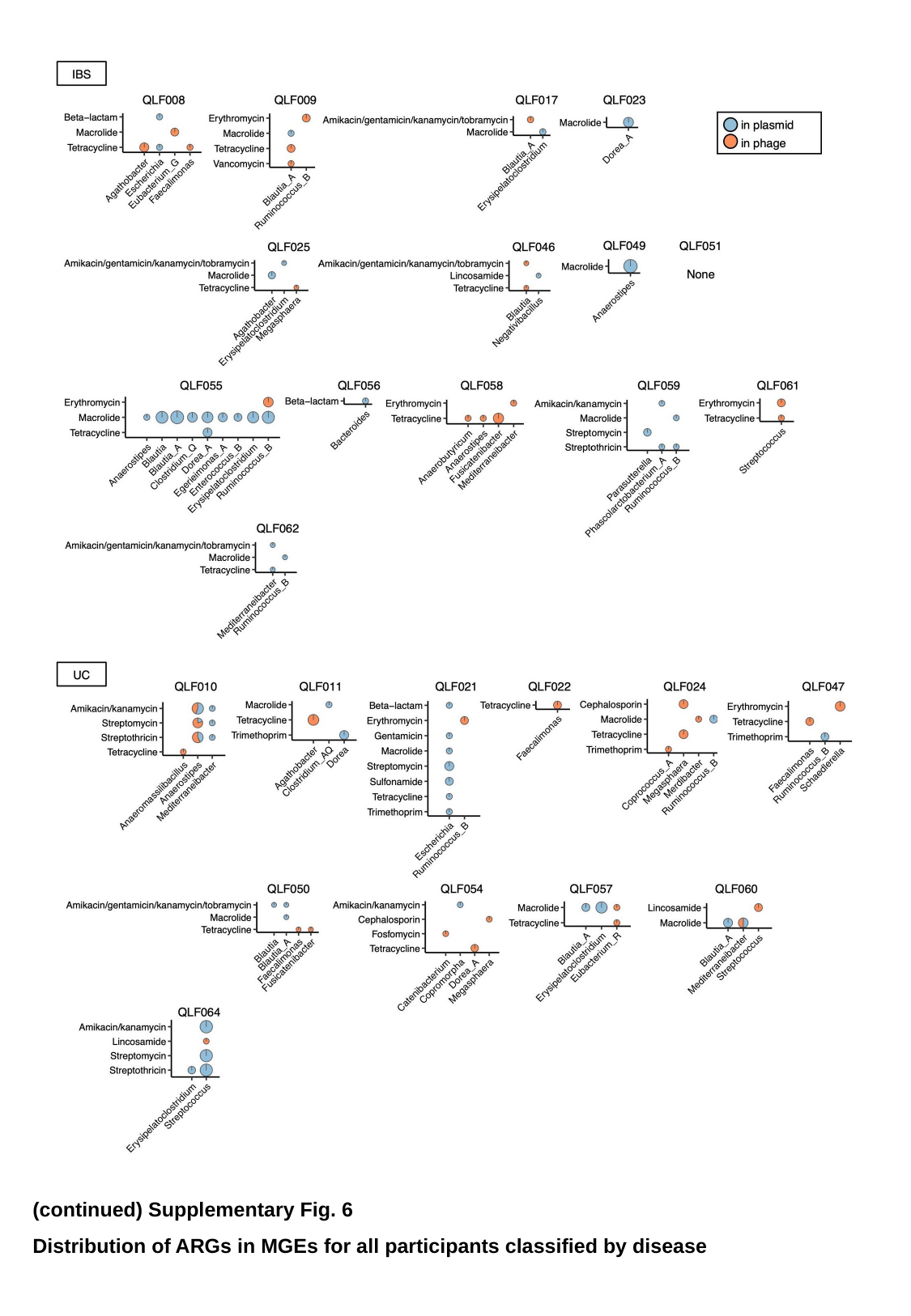

(continued) Supplementary Fig. 6
Distribution of ARGs in MGEs for all participants classified by disease
